# Contact prediction is hardest for the most informative contacts, but improves with the incorporation of contact potentials

**DOI:** 10.1371/journal.pone.0199585

**Published:** 2018-06-28

**Authors:** Jack Holland, Qinxin Pan, Gevorg Grigoryan

**Affiliations:** 1 Department of Computer Science, Dartmouth College, Hanover, NH 03755, United States of America; 2 Department of Biological Sciences, Dartmouth College, Hanover, NH 03755, United States of America; University of Michigan, UNITED STATES

## Abstract

Co-evolution between pairs of residues in a multiple sequence alignment (MSA) of homologous proteins has long been proposed as an indicator of structural contacts. Recently, several methods, such as direct-coupling analysis (DCA) and MetaPSICOV, have been shown to achieve impressive rates of contact prediction by taking advantage of considerable sequence data. In this paper, we show that prediction success rates are highly sensitive to the structural definition of a contact, with more permissive definitions (i.e., those classifying more pairs as true contacts) naturally leading to higher positive predictive rates, but at the expense of the amount of structural information contributed by each contact. Thus, the remaining limitations of contact prediction algorithms are most noticeable in conjunction with geometrically restrictive contacts—precisely those that contribute more information in structure prediction. We suggest that to improve prediction rates for such “informative” contacts one could combine co-evolution scores with additional indicators of contact likelihood. Specifically, we find that when a pair of co-varying positions in an MSA is occupied by residue pairs with favorable statistical contact energies, that pair is more likely to represent a true contact. We show that combining a contact potential metric with DCA or MetaPSICOV performs considerably better than DCA or MetaPSICOV alone, respectively. This is true regardless of contact definition, but especially true for stricter and more informative contact definitions. In summary, this work outlines some remaining challenges to be addressed in contact prediction and proposes and validates a promising direction towards improvement.

## 1 Introduction

Formation of tertiary structure in proteins is dependent on the establishment of close through-space interactions, often between amino-acid residues distant in sequence. Inter-residue contacts should impose constraints on evolutionary dynamics. Thus, mutations at contacting pairs are expected to be coupled in the evolutionary record. Such compensatory mutational coupling in evolutionarily related proteins enables statistical methods to infer which positions in a multiple sequence alignment (MSA) of structurally homologous proteins may be in contact. The idea of using predicted inter-residue contacts, discovered by analyzing MSAs, to aid in structure prediction has been around for decades [1], but has experienced a resurgence recently due to the massively increased amount of available sequence data [2–5]. Several investigators have now shown that the large sequence datasets available today enable much more robust contact predictions than their smaller counterparts [6–9]. However, any successful contact prediction model must avoid inferring spurious couplings [10]. Indeed, pairs of mutations can co-occur by chance or appear to couple due to phylogenetic biases, unrelated to maintaining structure [11]. Trying to determine which apparent correlations correspond to contacts has been approached from a variety of angles, such as enforcing maximum entropy to remove spurious indirect couplings [12], using probabilistic graphical models to learn correlations from sparse statistics [2], and estimating evolutionary distance relationships to determine the significance of correlations [13]. Impressive precision rates upwards of 90% have been reported for the most confident few predicted contacts [2], which can be enough for practical structure prediction [14–16].

Several challenges in contact prediction remain to be addressed, however. For instance, accuracy drops considerably when more than a few contacts are predicted [17]. Additionally, current methods require large numbers of sequences in the right range of homology that are unavailable in many practical scenarios [18]. But perhaps more importantly, the high reported prediction rates are in relation to fairly loose definitions of contact between two residues—for instance, any two atoms being within 8 Å of each other in any available structure belonging to the family in question [12] or any two Cβ atoms being within 8 Å [19]. This aids in achieving a high precision rates, but such loose definitions may not be optimal for the purpose of making predictions about structure.

A reasonable quality measure for a contact definition is the amount of information, per contact, contributed towards discriminating correct from incorrect structural models. Guided by this idea, we propose a new contact definition, termed *contact degree* (CD), and show that the knowledge of a single CD-based contact eliminates considerably more solution space in structure prediction than does knowledge of a contact defined via common distance-based criteria. On the other hand, we find that MSA-based contact prediction results in much lower precision for CD-based contacts as it does for traditional contact definitions. Thus, the remaining challenges in contact prediction are better revealed by adopting stricter definitions of contact that are ultimately more informative for structure prediction.

Motivated by these observations, and the need for both an informative contact definition and accurate prediction rates, we consider an additional source of information that can be used to supplement co-variation in contact prediction. In particular, we consider the fact that different amino-acid pairs have different *a priori* expectations of being in contact, based on observations in native proteins. These differential expectations are captured within so-called residue-level statistical contact potentials [20]. While contact potentials cannot encode all of the information required to fold a structure [21], they can be used to differentiate native structures from many varieties of decoys [22]. Thus, if a pair of MSA positions predicted to co-vary tends to be occupied by amino-acid pairs that do not score favorably by a residue-level contact potential, this should weaken our belief that the pair represents a true contact. On the other hand, if mutations at this pair of positions appear to compensate for each other in such a way as to produce consistently favorable contact potentials, this pair may be more likely to be a true contact. Based on this intuition, we propose a metric that combines a contact potential with a co-evolution score (from DCA or MetaPSICOV) and show it to improve the precision of both DCA and MetaPSICOV alone considerably.

The idea of using contact potentials in contact prediction has been put forth in recent work [19, 23–25]. For example, Jones *et al.* include contact potential values as one of the many features in their neural network for predicting contacts [19]. In the analysis of the EPSILON-CP method developed by [25], the mean contact potential energy is deemed an important feature in the neural net. However, to our knowledge, the isolated benefit of contact potentials towards improving contact prediction has not been studied extensively. Furthermore, it has been unclear to what extent the significant degradation in performance resulting from the utilization of more informative contact definitions can be mitigated by the incorporation of contact potentials. Here we show that the added benefit of incorporating contact potentials can be quite significant, especially in conjunction with contact definitions that are difficult to predict but highly informative. Further, we find that averaging contact potential values across all sequences of an MSA (for a given pair of positions) produces significantly higher improvements in performance. Thus, in summary, this work both points out the significant room for improvement that remains towards accurately predicting informative inter-residue contacts and proposes a route towards attaining such improvement.

## 2 Results

### 2.1 Contact definition and interpretation

**Fig 1:**
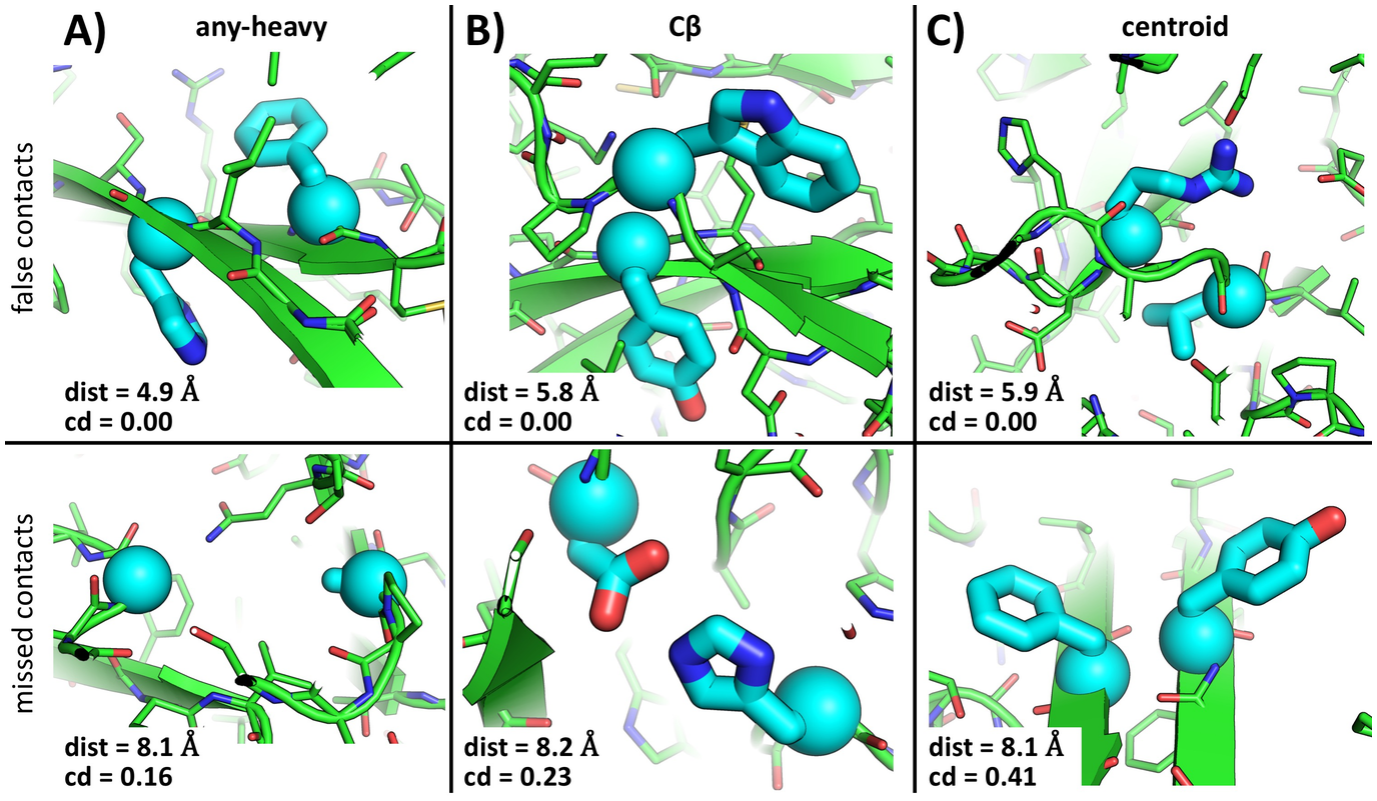
Distance-based contact definitions can flag unreasonable contact geometries or fail to capture position pairs likely to co-vary. **A)**, **B)**, and **C)** correspond to any-heavy, *C*_*β*_, and centroid-based contact definitions, respectively. The top row show examples where residue pairs that would be classified as contacting, on the basis of a rather strict distance cutoff in each case, do not appear to have immediate influence on each other. Whereas the bottom row demonstrates cases where a rather loose distance cutoff, in each case, would miss an apparent contact (i.e., a pair of positions likely to co-vary). The value of the corresponding distance metric, along with the contact degree value, are shown at the bottom of each panel. Residue pairs of interest are highlighted in thick cyan sticks, with their *Cα* atoms shown with spheres. The contacts shown in the top row correspond to position pairs (A126, A141), (A328, A344), and (V120, V128) from PDB structures 3JUM, 3JU4, and 1LM8 for **A)**-**C)**, respectively, and those in the bottom row correspond to position pairs (A55, A62), (C102, C201), and (B144, B153) from PDB structures 1JUH, 1JUH, and 4ACF for **A)**-**C)**, respectively. These illustrative cases were identified by manual inspection of a random set of 100 PDB structures. Molecular renderings created with PyMOL.

The best criterion for classifying a pair of residues as being in contact depends on the application—i.e., the meaning that a contact is interpreted to have. For many applications, including structure prediction and protein design, a reasonable interpretation of a contact would be a pair of residues that are capable of participating in a direct physical interaction in such a way as to have significant influence on each other’s amino-acid identities. Such an interpretation would be particularly well aligned with the goal of predicting contacts based on mutational co-variation. It follows then that spatial proximity should be an important but not the sole determinant of a contact. The opportunity to establish an interaction, as determined by the surrounding structural environment, should also be a contributor. Traditional distance-based contact definitions capture the former but not the latter factors. Fig 1 shows several examples of typical structural circumstances where a distance-dependent definition of contact does not agree with structural intuition. In particular, we consider three different commonly-used contact definitions: the one proposed by Morcos *et al.* in presenting the DCA method—i.e., two residues with at least one pair of non-hydrogen atoms within 8 Å of each other (hereafter referred to as the “any-heavy” definition) [12], the official CASP definition—i.e., two residues with Cβ (or Cα in the case of Glycine) atoms within 8 Å of each other (referred to as the “Cβ” definition) [19], and a definition based on a metric used in coarse-grained modeling—two residues with centroids within 6 Å of each other (referred to as the “centroid” definition) [26, 27]. The top row in Fig 1A–1C shows situations where each of these definitions, respectively, would classify as contacting position pairs that, by structural intuition, should not directly affect each other’s amino-acid identity; even with stricter thresholds than stated above. For example, in Fig 1A, the two positions involved are on opposite sides of a β-sheet. On the other hand, the bottom row in Fig 1A–1C demonstrates examples where each of the above definitions, respectively, would fail to classify as contacting residue pairs that would be expected to affect each other’s amino-acid identities and, therefore, would be expected to co-vary even with more generous cutoffs that those typically used.

In order to overcome these flaws, we propose a more structurally informative definition of a contact, based on the metric of a *contact degree*, which we have used in prior work [28, 29]. Rather than demarcate a contact based purely on distance, a contact degree considers all possible amino-acid and rotamer pair combinations for the position pair of interest and produces a value between 0 to 1 that represents the fraction of interfering rotamer pairs (i.e., those with non-hydrogen atoms within 3 Å of each other). More formally, the contact degree between two positions *i* and *j*, denoted *CD*_*i*,*j*_, is defined as follows:

$$\begin{array}{c} CD_{i,j}=\sum_{r_{i}\in R_{i}}\sum_{r_{j}\in R_{j}}C_{ij}\left(r_{i},r_{j}\right)\cdot P_{i}\left(r_{i}\right)\cdot P_{j}\left(r_{j}\right) \end{array}$$ (1)

Here, *R*_*i*_ is the set of every allowed rotamer from every amino acid at position *i* (based on some rotamer library) that does not clash with the backbone. $P_{i}\left(r_{i}\right)$ is the probability of rotamer *r*_*i*_ at position *i*, taken from the rotamer library and normalized to unity over all non-clashing rotamers at *i*. *C*_*ij*_(*r*_*i*_, *r*_*j*_) is unity if rotamer *r*_*i*_ placed at position *i* interferes with rotamer *r*_*j*_ placed at *j* (i.e., there are non-hydrogen atoms within 3 Å between the two rotamer side-chains) and zero otherwise. Thus, if none of the sterically possible rotamer pairs at the two positions interfere with each other, then *CD*_*i*,*j*_ = 0. At the other extreme, if all sterically possible rotamer pairs placed at *i* and *j* interfere, then *CD*_*i*,*j*_ = 1. To create a binary definition of contact, a cutoff *c* can be chosen so that all pairs of positions with a contact degree of at least *c* are considered to be in contact. In this study, we use *c* = 0.1. This gives an average of 4.1 contacts per residue, which is in line with our structural intuition.

Contact degree addresses the limitations of the distance-based definitions discussed above. Obviously, spacial proximity contributes to the criterion because position pairs far apart in space cannot host mutually interfering rotamers. However, the opportunity to interact is also accounted for by means of assessing contact via allowable rotamers (i.e., rotamers that are compatible with the surrounding structural environment). For example, all of the cases in Fig 1 are classified appropriately with a contact-degree cutoff of 0.1 (i.e., the top row is classified as non-contacting and the bottom row as contacting; corresponding contact degree and distance values are shown in each panel of Fig 1). As an added benefit, because contact degree does not rely on the sidechain coordinates of a structure, it is sequence independent. That is, one can assesses the possibility of a contact between two positions in a protein structural template, independent of the specific sequence associated with it (unlike, for example, with the centroid-based definition). This lends itself better to interpreting contacts as implying mutational co-dependence, especially within an evolutionary protein family.

### 2.2 Contact potential as a quality measure of contact definition

Given any geometric definition of inter-residue contact, one can derive a corresponding contact potential—a table of statistical pseudo-energies that reflect the relative propensity of different amino-acid types to be in contact within native-like protein structures [22, 30, 31]. We reasoned that a good quality metric for a contact definition would be the predictive power of the resulting contact potential. Of course, this is not the only quality metric, particularly given the fact that a contact potential alone is not sufficient to solve structure prediction [21]. Still, all else being equal, if the contact potential emergent from one contact definition systematically outperforms that emergent from another definition, it would seem reasonable to conclude that the former contact definition is better. Indeed, if a particular definition often classifies as contacting residue pairs that, in reality, do not significantly interact or influence each other, the resulting contact potential should have little meaning or predictive power. A similar argument would apply if a particular definition fails to classify many of the truly mutually influencing residues as contacting.

To evaluate the quality of our CD-based contact definition, we set out to compare the contact potential emergent from it relative to potentials emergent from several commonly-used distance-based contact definitions (see Table 1). To isolate just the effect of the contact definition, we used the same simple reference-state model in all cases. This model assumes random redistribution of amino acids among contacts, such that the statistical potential associated with the contact between amino acids *a* and *b* is:

$$\begin{array}{c} E\left(a,b\right)=-\log\left(\frac{N_{c}\left(a,b\right)}{\left(1+I_{a,b}\right)f\left(a\right)f\left(b\right)N_{c}}\right) \end{array}$$ (2)

**Table 1:** Contact definitions.

Name	Superscript	Description
CD-based	CD	contact degree greater than or equal to 0.1
any-heavy	1	at least one pair of non-hydrogen atoms within 8 Å of each other
Cβ	2	Cβ (or Cα in the case of Glycine) atoms within 8 Å of each other
centroid	3	residue sidechain centroids within 6 Å of each other

**Fig 2:**
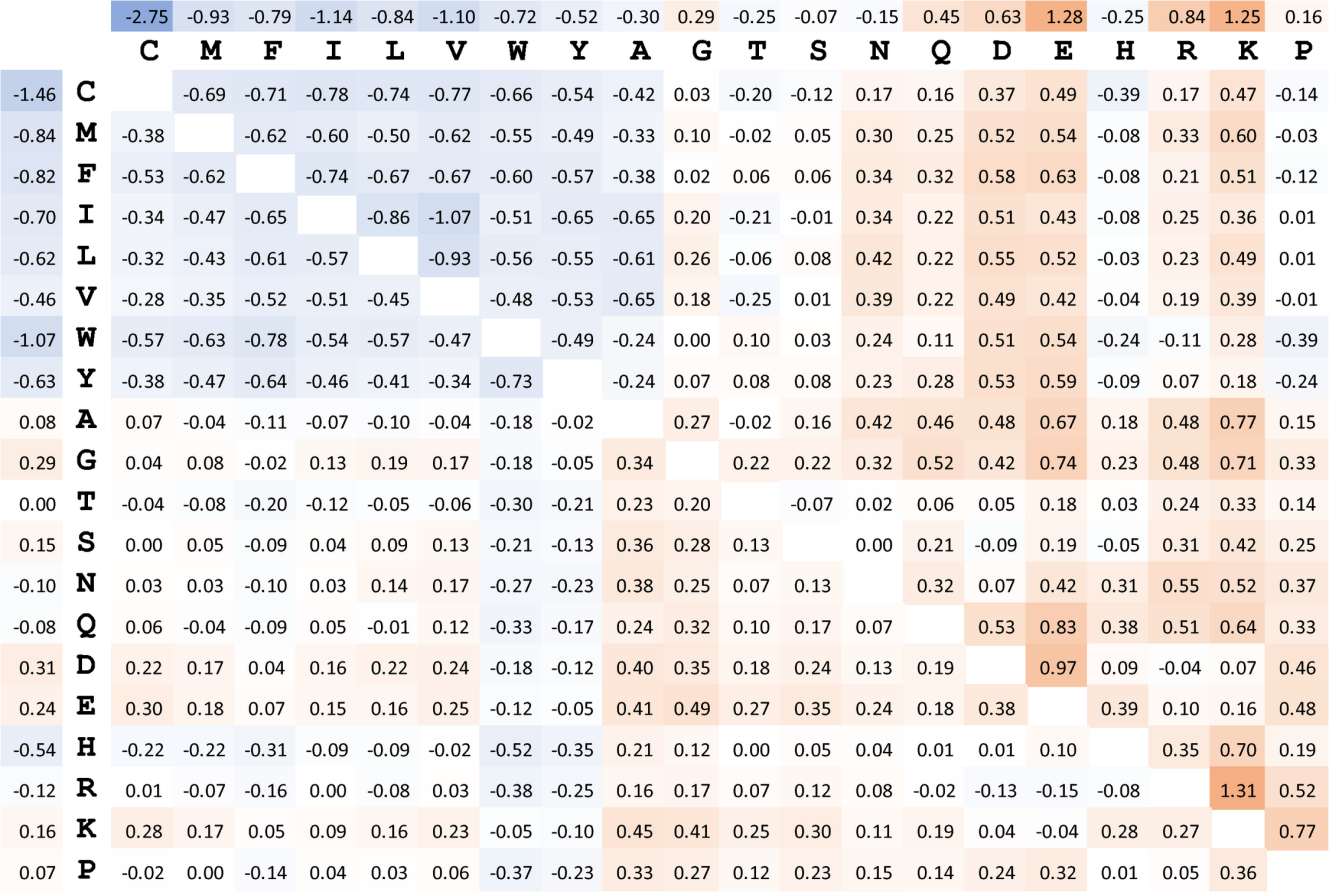
Statistical contact potential values for the CD-based definition of contact (upper right triangle and upper row for hetero- and homo-typic interactions, respectively) and the looser any-heavy-based definition (lower left corner and left column for hetero- and homo-typic interactions, respectively). Cells are colored blue to red in ascending order of statistical energies.

Here *N*_*c*_(*a*, *b*) is the number of observed contacts between *a* and *b*, *f*(*a*) is the frequency of amino acid *a* in the database, *N*_*c*_ is the total number of observed contacts (for all amino-acid pairs), and *I*_*a*,*b*_ is an indicator variable that evaluates to unity if *a* and *b* are different and to zero otherwise. As the structural database, we used the PISCES set prepared by the Dunbrack lab that included 8106 structures, each with a maximum resolution of 2.2Å culled at 30% sequence identity [32]. Fig 2 shows the pairwise contact-potential values for the CD-based and any-heavy-based potentials, which are generally well correlated (*R* = 0.81), but with non-negligible differences. For example, the mean absolute energy for the CD-based definition is 0.39, higher than the corresponding value of 0.23 for the any-heavy-based definition. This means that the degree of over/under-representations in amino-acid identities at contacting positions is generally higher for the CD-based definition, suggesting that it captures more of the underlying structural determinants of a true interaction. The same is also true when comparing the CD-based definition with Cβ and centroid definitions, which have mean absolute energies of 0.17 and 0.35, respectively. Hereafter, we will refer to the CD-based, any-heavy-based, Cβ-based, and centroid-based contact potentials as *E*_*CD*_, *E*_1_, *E*_2_, and *E*_3_, respectively (see Table 1).

### 2.3 Comparison of contact potentials via decoy discrimination

To evaluate the predictive performance of each contact potential, we turned to decoy discrimination. A common benchmark experiment for structure-prediction scoring functions, it tests whether the correct native (or a native-like) protein structure for a given sequence can be identified from a set that additionally includes incorrect/decoy structures. Specifically, we used two commonly employed decoy sets: the I-TASSER Decoy Set-II generated by the Zhang lab [33] and the Rosetta decoy set by the Baker lab [34]. These have been broadly used to test a variety of scoring methods [35–42]. The decoys in these two datasets were generated differently, and therefore represent different test cases for a scoring function. I-TASSER decoys were generated by refining I-TASSER *ab initio* predictions with the OPLS-AA force field in order to remove clashes and optimize torsion angles. The Rosetta decoys were generated by swapping native backbone dihedral angles with random ones from other native structures, filtering out structures with overly high radii of gyration or those with heavy atom clashes. The I-TASSER set contains 56 proteins, with 300-500 decoys for each, and the Rosetta set has 59 proteins with 100 decoys for each.

For each protein, the native structure and all of its decoys were scored using each potential. To evaluate performance, the rank of the native structure based on its score was determined for each protein in the sets. A rank of 1 means that the native received the most favorable score, whereas higher ranks indicate that some decoy structures scored better than the native. Table 2 shows the performance on the I-TASSER Decoy Set-II [33]. Among the four contact potentials considered, *E*_*CD*_ assigns the lowest rank to the native structure (or is tied for the lowest rank) in 37 cases, whereas *E*_1_, *E*_2_, and *E*_3_ do so in 4, 10, and 10 cases, respectively. Overall, the ranks assigned by *E*_*CD*_ are well below those for all other potentials, and these differences in performance are highly statistically significant (see Table 2). Table 3 shows the performance on the Rosetta decoy set [34]. In this case, *E*_*CD*_ assigns the lowest rank to the native structure (or is tied for the lowest rank) in 27 cases, whereas the same is true for *E*_1_, *E*_2_, and *E*_3_ in 7, 17, and 25 cases, respectively. The Rosetta decoy set appears to be a significantly simpler set than the I-TASSER one for all contact potentials, so differences in performance are less pronounced. Thus, although *E*_*CD*_ numerically outperforms all other potentials here as well, the difference is statistically significant only in comparison with *E*_1_, whereas *E*_2_ and *E*_3_ perform similarly to *E*_*CD*_ (see Table 3).

**Table 2:** Decoy-discrimination performance of *E*_*CD*_, *E*_1_, *E*_2_, and *E*_3_ potentials (in columns CD, any-heavy, CB, and centroid, respectively) on the the I-TASSER II decoy set. Shown is the rank of native structure, in each sub-set, by the corresponding contact potential. The ranking of natives by *E*_*CD*_ is significantly better than the rankings using the other potentials, with the p-values from the Friedman test being 7.9 ⋅ 10^−10^, 1.3 ⋅ 10^−5^, and 4.5 ⋅ 10^−5^ when comparing *E*_*CD*_ with *E*_1_, *E*_2_, and *E*_3_, respectively.

Name	CD	any-heavy	*C*_*β*_	centroid	Name	CD	any-heavy	*C*_*β*_	centroid
1abv_	100	221	366	320	1mkyA3	87	267	234	151
1af7__	13	492	101	101	1mla_2	17	103	194	125
1ah9_	392	450	212	152	1mn8A	196	392	373	503
1aoy_	147	397	474	445	1n0uA4	171	269	266	277
1b4bA	3	322	52	6	1ne3A	76	498	537	503
1b72A	392	486	512	534	1no5A	2	36	2	84
1bm8_	3	208	10	40	1npsA	214	385	363	365
1bq9A	8	389	298	7	1o2fB_	4	248	246	19
1cewI	137	438	359	243	1of9A	1	507	432	31
1cqkA	2	282	23	76	1ogwA_	240	333	243	192
1csp_	220	305	195	255	1orgA	3	65	4	1
1cy5A	48	274	227	249	1pgx_	379	157	452	349
1dcjA_	72	2	289	69	1r69_	17	2	208	110
1di2A_	226	17	225	198	1sfp_	61	309	7	211
1dtjA_	18	284	90	282	1shfA	67	502	335	362
1egxA	83	156	20	13	1sro_	85	476	6	86
1fadA	95	391	337	430	1ten_	11	258	256	219
1fo5A	145	289	235	334	1tfi_	264	234	94	103
1g1cA	32	290	135	35	1thx_	4	228	40	6
1gjxA	32	474	283	256	1tif_	12	422	367	486
1gnuA	10	467	441	238	1tig_	201	478	466	397
1gpt_	56	383	316	343	1vcc_	9	550	414	398
1gyvA	12	229	5	60	256bA	335	445	336	335
1hbkA	172	265	234	178	2a0b_	219	234	221	218
1itpA	376	473	445	250	2cr7A	102	257	101	101
1jnuA	6	236	11	161	2f3nA	274	455	442	274
1kjs_	240	270	176	339	2pcy_	249	324	249	354
1kviA	455	475	298	540	2reb_2	45	91	309	337
**Median**	79.5	297.5	244.5	228.5					

**Table 3:** Decoy-discrimination performance of *E*_*CD*_, *E*_1_, *E*_2_, and *E*_3_ potentials (in columns CD, any-heavy, CB, and centroid, respectively) on the Rosetta decoy set. Shown is the rank of native structure, in each sub-set, by the corresponding contact potential. The ranking of natives by *E*_*CD*_ is significantly better than ranking by the all-heavy potential (*E*_1_), and potentials *E*_2_ and *E*_3_ performing similarly to *E*_*CD*_ (Friendman test p-values are 10^−7^, 0.17, and 0.78, respectively).

Name	CD	any-heavy	*C*_*β*_	centroid	Name	CD	any-heavy	*C*_*β*_	centroid
1a19	8	26	14	22	1kpe	13	48	10	1
1a32	50	101	92	27	1lis	63	100	15	14
1a68	63	101	35	12	1lou	12	87	27	32
1acf	1	35	10	10	1nps	4	12	11	17
1ail	4	20	3	16	1opd	2	6	6	22
1aiu	61	101	67	64	1pgx	5	1	69	19
1b3a	16	80	48	38	1ptq	7	101	60	10
1bgf	35	76	15	11	1r69	38	1	54	37
1bk2	13	74	13	3	1rnb	1	18	1	22
1bkr	8	39	12	1	1scj	35	30	59	20
1bm8	1	34	10	1	1shf	25	68	22	38
1bq9	18	37	10	9	1ten	1	1	1	1
1c8c	15	49	34	13	1tig	5	48	2	25
1c9o	53	99	36	45	1tul	7	14	10	1
1cc8	29	35	8	17	1ubi	61	84	48	41
1cei	40	12	17	5	1ugh	4	46	33	57
1cg5	29	59	6	15	1urn	2	50	20	2
1ctf	53	1	14	4	1utg	100	101	101	100
1dhn	1	54	6	1	1vcc	6	94	20	9
1e6i	7	96	1	17	1vie	25	40	36	62
1elw	16	1	70	87	1vls	65	62	13	60
1enh	67	93	51	62	1who	1	10	1	1
1ew4	1	22	2	4	256b	62	1	28	76
1eyv	2	17	10	9	2acy	1	13	1	5
1fkb	1	14	4	1	2chf	23	87	36	72
1fna	19	33	27	14	2ci2	8	100	37	73
1gvp	6	76	41	15	2tif	1	1	1	1
1hz6	16	32	10	11	4ubp	1	33	1	1
1ig5	21	27	1	90	5cro	74	55	43	13
1iib	23	94	27	14					
**Median**	13	40	15	15					

Because the only difference between these potentials is the definition of contact (the reference state is kept the same), the above results strongly suggest that CD is a more informative criterion for determining residue interactions. Thus, it would appear to be more advantageous for structural modeling to predict contacts defined via CD than the looser distance-based criterion. To test this claim more directly, we measured the amount of information contributed by each native contact to decoy discrimination. That is, we asked what fraction of decoys are eliminated (on average) by the knowledge of a single contact in the native structure. We found that for the CD-based definition, an average contact eliminates 64% of the Rosetta decoys whereas this fraction is 48%, 48%, and 63% for the any-heavy-, Cβ-, and centroid-based definitions, respectively. Similarly, on average a CD-based contact eliminates 72% of the I-TASSER decoys compared to 47%, 44%, and 66%, respectively, for the other three contact definitions. This shows that it would be more advantageous, for the purposes of structure prediction, if evolutionary MSA-based methods predicted contacts under the CD-based definition.

### 2.4 Contact prediction using different contact definitions

**Fig 3:**
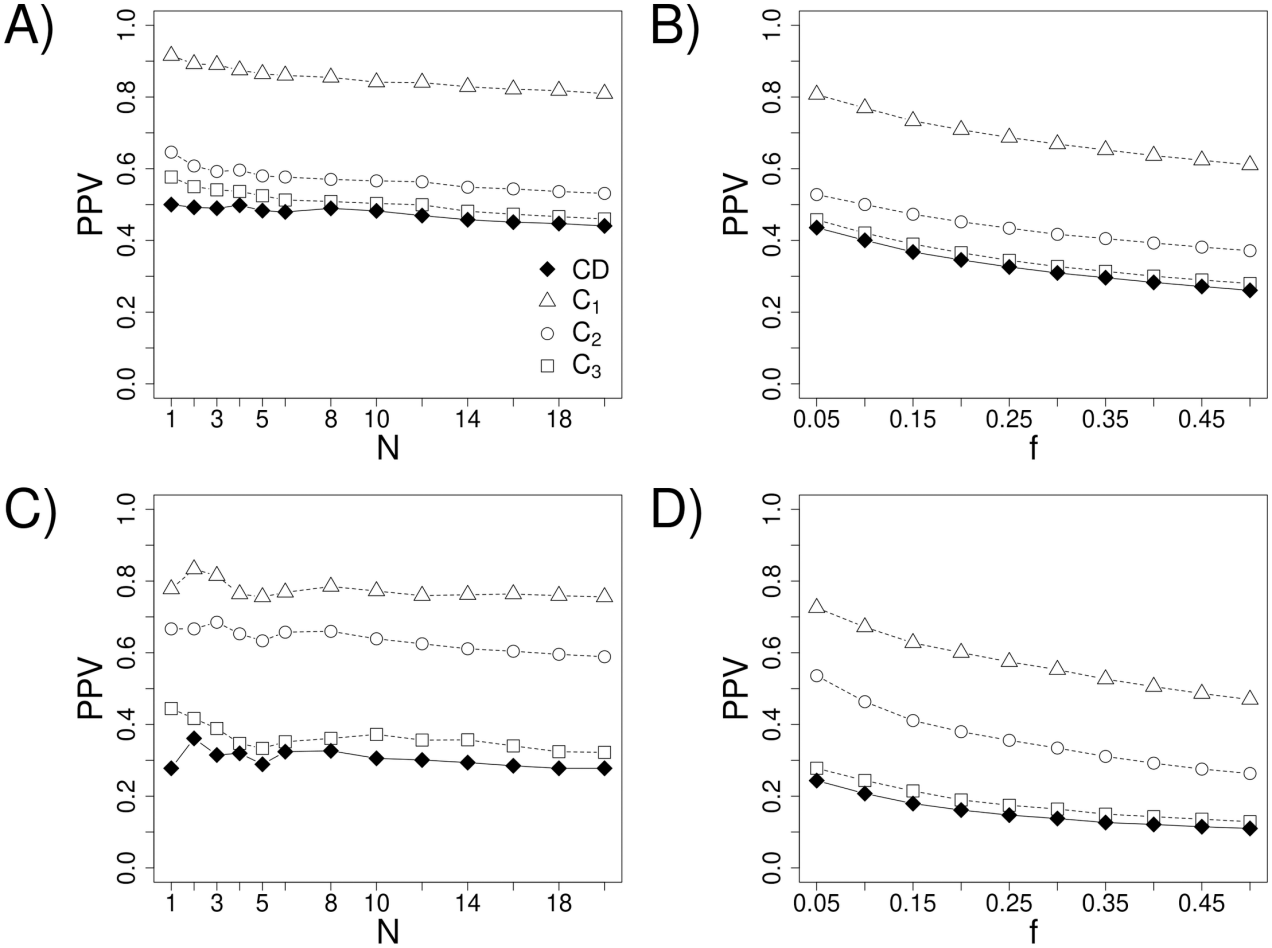
Average PPV of contact prediction as a function of the number (*N*) or fraction (*f*) of predictions. Predictions labeled by *CD* refer to predictions when contacts are defined by contact degree and those labeled by *C*_1_, *C*_2_, and *C*_3_ refer to predictions when contacts are defined by the other three definitions (see Table 1 for details). (A, B) Predictions of DCA on the Pfam dataset. (C, D) Predictions of MetaPSICOV on the CASP12 dataset.

We next asked how well the more valuable CD-based contacts are predicted from MSAs using the principle of co-evolution. As representative methods, we used 1) the Direct Coupling Analysis (DCA) approach by Morcos *et al.* [12], which has aided a number of structure prediction tasks [43–46]; and 2) MetaPSICOV by Jones *et al.*, a state-of-the-art consensus method that combines three different co-evolution calculations (PSICOV [47], mean-field DCA [48], and CCMpred [49]) with other features (e.g., predicted secondary structure, solvent accessibility, and others) into a neural network. MetaPSICOV has been among the best performers in the contact prediction category of recent CASP competitions [19, 50]. In the DCA method, the direct information (DI) metric computed for all position pairs in an MSA is used to order the likelihood that each corresponds to a true contact, with a higher DI indicating a more likely contact. In MetaPSICOV’s case, the output of the neural network produces a value between 0 and 1 termed the *precision score*, with a higher value indicating a more likely contact. Fig 3 shows the performances of DCA and MetaPSICOV in the context of either the CD-based or the looser distance-based definitions of true contact. Shown is the positive predictive value (PPV) as a function of either the number of pairs predicted as contacting (*N*, Fig 3A and 3C) or the length-normalized number (i.e., fraction) of predicted contacts (*f*, Fig 3B and 3D), respectively.

Though different datasets are used to evaluate DCA and MetaPSICOV in Fig 3 (thus, absolute results are not directly comparable between the two; see Methods), in all cases, the performance is lowest with the CD-based contact definition. Thus, although CDs are more informative, they appear harder to predict correctly. In general, unsurprisingly, contacts by looser criteria appear easier to predict. Indeed, ~20%, ~10%, and ~6% of position pairs are classified as contacting by the the any-heavy, Cβ, and centroid definitions, respectively, whereas only ~4% are in contact by the CD-based definition. This is consistent with contact prediction performance monotonically increasing in the order of CD, centroid, Cβ, and any-heavy contact definitions (see Fig 3). Based on the above contact frequencies, a randomly chosen position pair is, respectively, ~5.0, ~2.5, and ~1.5 times more likely to be a true contact by the any-heavy-, Cβ-, and centroid-based definition than by the CD-based one. On the other hand, the PPV for predicting CD-based contacts is reduced relative to that for other definitions by significantly lower fractions (see Fig 3A). Thus, it would seem that predicting CD-based contacts may still provide more information. Notably, the greatest discrepancies in performance among the different definitions of contact occur for long-range contacts, defined as those with a sequence separation of at least 23 (S1 Fig). Given that long-range contacts tend to constrain the possible structure more than short-range contacts, these performance discrepancies are particularly important to address.

The above results suggest that contact degree captures useful information about structure, more so than other contact definitions, but the considerably lower precision of predicting it is not desirable, so we next seek ways of improving it.

### 2.5 A statistical contact potential aids in contact prediction

A statistical contact potential provides a convenient line of additional evidence towards predicting contacts, because it quantifies the *a priori* expectation that any two amino acid types would be in contact. Looking at a particular pair of positions (*i*, *j*) in an MSA, we can ask whether the amino-acid pairs found at these positions tend to correspond to favorable or unfavorable contact-potential values. Qualitatively, if the former is the case, this should strengthen our belief that (*i*, *j*) is a true contact, while the latter case would weaken this belief. To capture this quantitatively, one could (for example) look at the average value of a contact potential across all amino acid pairs at (*i*, *j*) in the MSA, which we will denote ${\hat{E}}^{i,j}$. This metric could then be used in combination with co-evolution scores (e.g., DI or precision score for DCA or MetaPSICOV, respectively) to make a call about a particular position pair. To test this concept, we propose a simple empirical metric:

$$\begin{array}{c} S^{i,j}=S^{i,j}\left(1-\frac{{\hat{E}}^{i,j}}{S_{max}}\right) \end{array}$$ (3)

where *S*^*i*,*j*^ is the MSA-based co-evolution score for the position pair (*i*, *j*) and *S*_*max*_ is the maximal value of the former for any pair of positions in the given alignment. The reasoning behind this combination is that contact potential values are on a fixed scale, whereas we have empirically found co-evolution scores to vary considerably from case to case, depending significantly on the depth and other properties of the MSA. Dividing ${\hat{E}}^{i,j}$ by *S*_*max*_ then serves to normalize the two metrics with respect to each other, across different MSAs. The negative sign in front of ${\hat{E}}^{i,j}$ reflects the fact that negative potential values correspond to favorable cases and the product ensures that *S*^*i*,*j*^ and ${\hat{E}}^{i,j}$ jointly contribute towards scoring a potential contact. Note that much more sophisticated combinations of *S*^*i*,*j*^ and *E*^*i*,*j*^ are possible. In fact, MetaPSICOV includes the value of a statistical contact potential as one of the features that go into its neural network model [19]. However, our focus here is to establish and quantify the value of using contact potentials to augment co-evolution scores, under different contact definitions, so we chose a simple functional form for ease of interpretation.

**Fig 4:**
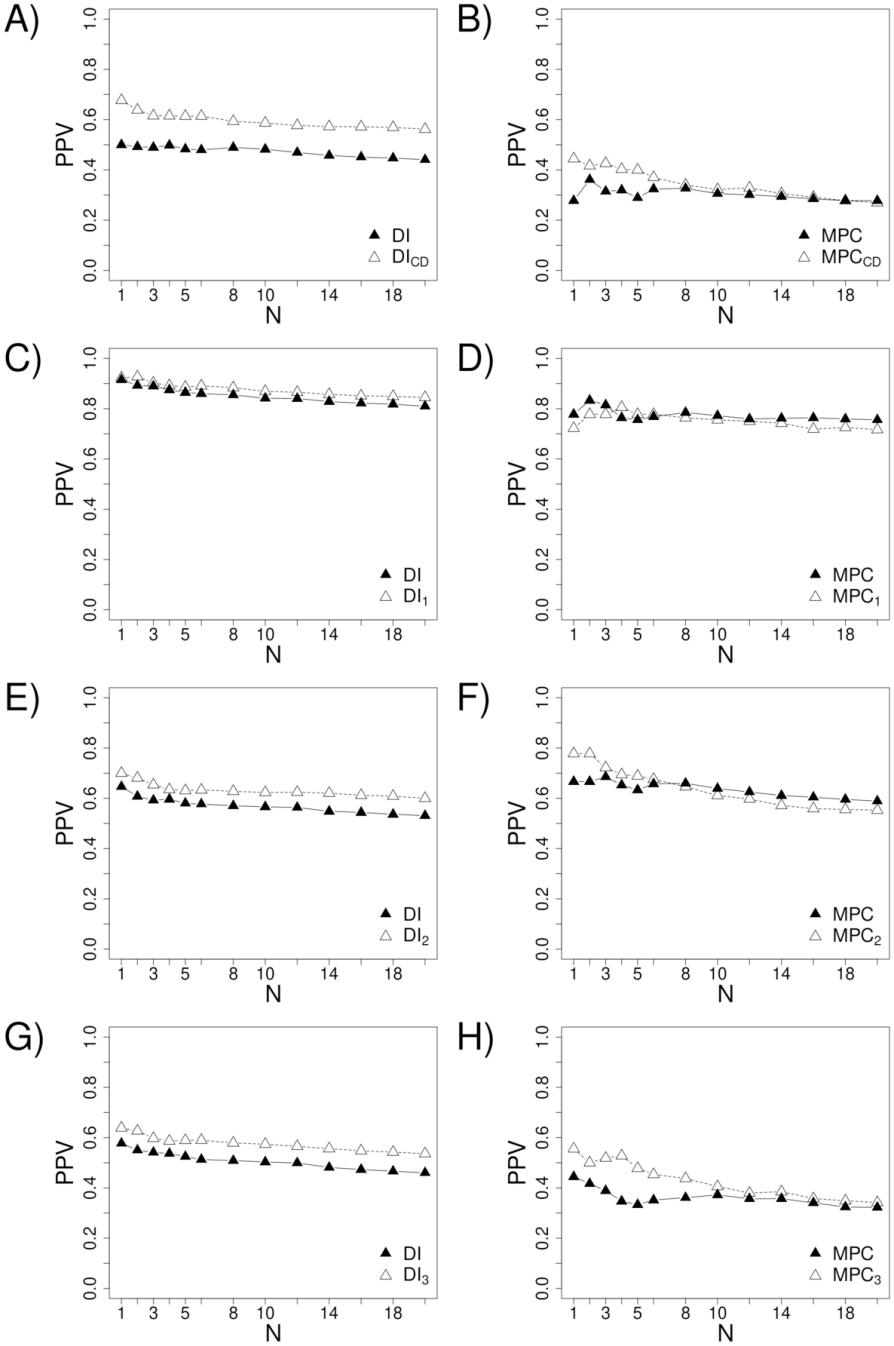
The effects of incorporating a contact potential into contact prediction. In plots A, C, E, and G, *DI* refers to predictions made using direct information alone. In plots B, D, F, and H, *MPC* refers to MetaPSICOV’s predictions alone. *DI*_*CD*_ and *MPC*_*CD*_ respectively refer to DI and MPC’s predictions augmented by contact degree (see Eq (3)). Similarly, for *n* ∈ {1, 2, 3}, *DI*_*n*_ and *MPC*_*n*_ respectively refer to DI and MPC’s predictions augmented by contact definition *n*.

We consider each of the contact definitions discussed above and derive four corresponding augmented *S* metrics, $S_{CD}^{i,j}$ and $S_{1}^{i,j}$, $S_{2}^{i,j}$, and $S_{3}^{i,j}$. Fig 4 compares the performance of these combined metrics with that of unadjusted *S* towards predicted the corresponding contact types (i.e., how well $S_{CD}^{i,j}$ predicts CD-based contacts and how well each distance metric predicts the corresponding distance-based contacts). Encouragingly, the PPV for predicting CD-based contacts increases by as much as ~18% and ~12% for the first few predictions using DCA and MetaPSICOV, respectively (Fig 4A and 4B). The performance also increases for the distance-based contact definitions (Fig 4C–4H). These increases are smaller that with CD-based contacts, with the exception of the centroid definition in conjunction with MetaPSICOV improving PPV by a comparable amount (~14% for the first few contacts). The PPV using the any-heavy definition is close to perfect—over 90% for the first few contacts—but incorporating the any-heavy potential still systematically improves the performance, demonstrating the general benefit of incorporating a contact potential.

**Fig 5:**
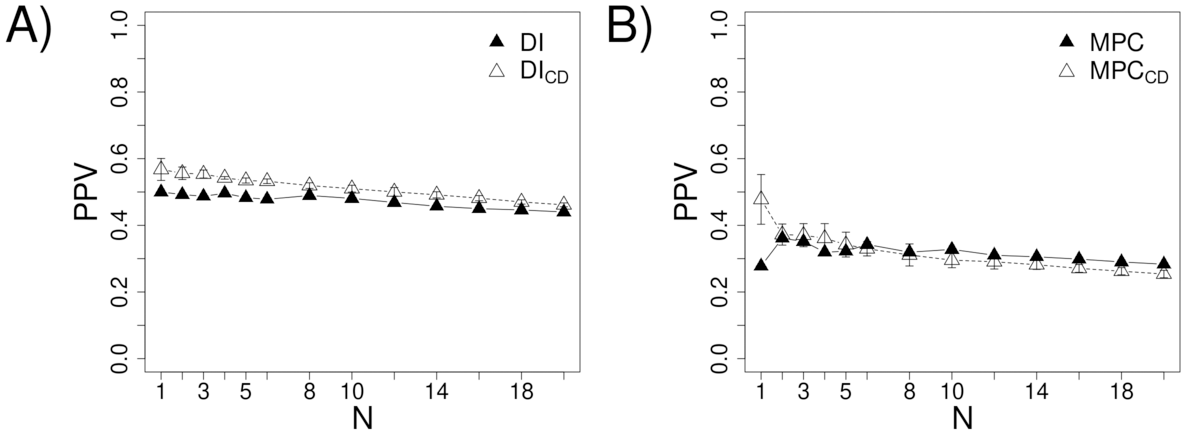
Contact predictions made using (A) DCA and (B) MetaPSICOV alone are compared against predictions that combine co-evolution scores with the CD-based contact potential energies from a single randomly-chosen sequence in each alignment. This procedure was repeated five times. Each point displayed corresponds to the mean PPV and the error bars show the standard deviation.

We next ask whether there is benefit in averaging the statistical contact potential values over all sequences of an MSA. That is, we ask whether comparable performance improvements are observed when the contact potential is computed only in the context of a single sequence (e.g., the sequence for which contacts are being predicted). To that end, Fig 5 shows the performance improvement (averaged over five trials) when contact-potential energies are calculated in the context of only a single sequence randomly selected from the corresponding MSA. For DCA applied to the Pfam dataset (see Methods) incorporating these energies systematically improves the PPV (Fig 5A). For MetaPSICOV applied to the CASP12 dataset (see Methods) the improvement is marginal at best (in fact, the performance drops slightly for larger *N*; Fig 5B). This suggests that averaging contact potential values over the MSA does provide a significant benefit over evaluation in the context of a single sequence (compare Figs 4A and 5A). On the other hand, average contact-potential values on their own do not provide sufficient information for effective contact prediction (e.g., see S2 Fig for the performance of the CD-based contact potential on the DCA dataset).

We further test how the diversity of predicted contacts changes when different contact potentials are combined with co-evolution scores. Higher contact diversity is desirable because if a method’s predicted contacts cover many regions in the contact map, each predicted contact can independently restrain the possible structures the sequence might fold into. To assess contact diversity, we adopted the definition used by He *et al.*, wherein the contact map of each target was divided into a 10 x 10 grid of equal-sized regions and the diversity *D* was quantified as the Shannon entropy of the distribution of the top *N*/2 contacts over these regions (where *N* is the length of the MSA) [51]:

$$\begin{array}{c} D=-\sum_{i}^{100}p_{i}\log_{2}p_{i} \end{array}$$ (4)

Here, *p*_*i*_ is the fraction of contacts that fall within region *i*. Table 4 shows the mean *D* over all targets when contacts are either ranked by co-evolution scores alone or by hybrid scores that combine the different contact potentials. Clearly, for both DCA and MetaPSICOV, diversity increases upon adding all contact potentials, but it increases the most when the CD-based contact potential is added.

**Table 4:** The effect of incorporating contact potentials on contact diversity. Contact diversity was quantified by applying Eq (4) to the top *N*/2 contacts in each alignment and then averaging over every alignment in the dataset (first row: DCA on the Pfam dataset; second row: MetaPSICOV on the CASP12 dataset, see Methods), where *N* is the length of an alignment. The “alone” column contains the diversities when no contact potential is applied (that is, when DCA or MetaPSICOV scores alone are used to rank contacts). The remaining columns contain the diversities resulting from ranking contacts by hybrid scores that combine the corresponding co-evolution score and a contact potential (based on the four contact definitions in Table 1, respectively).

	alone	with *E*_*CD*_	with *E*_1_	with *E*_2_	with *E*_3_
**DCA**	3.36	3.67	3.51	3.48	3.61
**MetaPSICOV**	3.38	3.65	3.54	3.49	3.61

## 3 Discussion

In this study we show that contact prediction performance depends critically on the underlying geometric definition of a contact. The previously reported high prediction rates have relied on relatively loose, distance-based definitions of contact. The definitions tested in this study—any heavy atoms within 8 Å, Cβ atoms within 8 Å, and centroid pseudoatoms within 6 Å– respectively classify ~20%, ~10%, and ~6% of the residue pairs in a protein as contacting. Though this aids in achieving a high positive predictive rates, the looseness comes at the expense of information contributed towards structure prediction. This is evident when comparing these contact definitions to a stricter one we propose, based on the quantity of contact degree (CD, Eq (1)). Indeed, only ~4% of position pairs are classified as contacting based on CD (with the cutoff of 0.1 used throughout this study) and a single CD-based contact eliminates 5, 2.5, and 1.5 times more decoy structures than a contact defined by the any-heavy, Cβ, and centroid definitions, respectively. Also, a statistical contact potential corresponding to the CD-based contact definition exhibits a significantly better performance in decoy discrimination than do contact potentials derived from distance-based contact definitions.

Though more informative, CD-based contacts are also harder to predict (see Fig 3). Encouragingly, however, we show that combining the co-evolution score of a given residue pair with the statistical contact potential energy for the pair, averaged over all sequences in the MSA, results in a significantly more predictive metric. The performance boost is particularly pronounced in the prediction of CD-based contacts. For example, the CD-based potential increases the precision of the DCA method by ~18% for the first few contacts (see Fig 4A). Such a performance increase is highly relevant given that the knowledge of only a few of contacts is often sufficient to aid structure prediction [52].

While the performance improvements were largest for CD-based contacts, incorporating a contact potential improved performance for every definition of contact using both methods, with the exception of the Cβ-based potential not improving the performance of MetaPSICOV. Notably, of the three distance-based contact definitions we have considered, the centroid-based definition exhibits considerable advantages: 1) it performs best (or tied for best) in decoy discrimination (see Tables 2 and 3), 2) contact-prediction improvement resulting from the incorporation of its corresponding contact potential is the highest (see Fig 4H), 3) it eliminates the highest fraction of decoys based on a single contact, and 4) it leads to the highest contact diversity increase when augmenting a co-evolution score (see Table 4). It can be argued that these advantages, to some extent, are a result of the centroid-based definition using more information–i.e., the location of the side-chain. Indeed, side-chains positions must be known (or appropriately modeled) to even apply this definition of a contact. On the other hand, the CD-based definition achieves better performance in all of the above criteria without requiring side-chain information. Possible side-chain positioning is accounted for explicitly within the CD calculation procedure itself, in a sequence independent manner, resulting in a contact definition that can be applied to full-atom or backbone-only models alike.

## 4 Methods

### 4.1 Contact degree

CDs were calculated according to Eq (1) using the 2010 backbone-dependent Dunbrack rotamer library [53]. Rotamers were labeled as clashing with the backbone (and removed from consideration) if at least one non-hydrogen atom in the rotamer sidechain was within 2.0 Å of any non-hydrogen backbone atom of the structure (except its own backbone). ConFind, a program that computes CDs, can be found at http://www.grigoryanlab.org/confind/.

### 4.2 Decoy discrimination

The I-TASSER II decoy set was downloaded from https://zhanglab.ccmb.med.umich.edu/decoys/decoy2.html [33]. The Rosetta decoy set was downloaded from https://zenodo.org/record/48780#.WqAU-HWnFhF [54].

### 4.3 DCA

As described by Morcos *et al.*, 131 protein families were selected from Pfam’s homologous sequence datasets based on the number of non-redundant sequences, fraction of sequences belonging to bacterial organisms, and the availability of high quality PDB structures [12] (see S1 Data for the accession number and sequence range of each sequence in each family’s alignment). This resulted in 856 corresponding PDB structures. DI for all residue pairs was calculated using Matlab code obtained from Dr. Morcos (see S1 Script for this script). To map the 856 PDB structures to their Pfam families, each PDB sequence was compared against all sequences in all of the above Pfam families. To account for point mutations introduced in PDB structures, a sequence-to-structure match was established if the sequence similarity was at least 95%. If no sequence was found to be a match for a particular PDB structure, the sequence that gave the highest sequence similarity score was considered as the match. In this way, each PDB structure in the list was mapped onto at least one of the 131 Pfam families. The MSAs and structures used for this analysis are exactly as those used in the original study, so the results in Fig 3A for the loose contact definition reproduce the PPVs reported in that work.

### 4.4 MetaPSICOV

To evaluate MetaPSICOV’s contact prediction, the sequences of each CASP12 target listed in Table 1 in Buchan *et al.* were submitted to the MetaPSICOV server (http://bioinf.cs.ucl.ac.uk/MetaPSICOV/) and the precision scores were extracted from the Stage 2 results [50]. Because not all CASP12 target sequences have publicly available structures, which are needed to determine which pairs of positions are in contact, only those sequences with corresponding PDB entries were considered, resulting in 19 sequences. Each sequence’s PDB ID was taken from the CASP website (http://predictioncenter.org/casp12/targetlist.cgi) and the corresponding PDB file was downloaded from the PDB. To acquire the alignments used to produce MetaPSICOV’s precision scores, MetaPSICOV was downloaded from http://bioinfadmin.cs.ucl.ac.uk/downloads/MetaPSICOV/ and run locally. Due to technical difficulties, the alignment for target T0918 could not be computed, resulting in a dataset of 18 sequences: T0859, T0862, T0863, T0864, T0866, T0868, T0869, T0870, T0886, T0892, T0896, T0897, T0898, T0900, T0904, T0941, T0943, T0945.

### 4.5 Contact potential

See S2 Data for the list of PDB IDs and chains comprising the dataset that the contact potentials were constructed from. See S3 Data for CSV files containing the energies of each contact potential.

### 4.6 Contact definitions

Contacts in each structure were identified using either the CD-based metric, with a cutoff of 0.1, or one of the three distance-based metrics specified in Table 1, *C*_1_, *C*_2_, and *C*_3_. For *C*_1_—“any-heavy”—a pair of positions was considered in contact if at least one non-hydrogen atom from the residue at one position was less than 8 Å of one non-hydrogen atom from the residue at the other position, backbone atoms included. For *C*_2_—“Cβ”—a pair of positions was considered in contact if the Cβ atom from one position was less than 8 Å from the Cβ atom from the other position. For *C*_3_—“centroid”—a pair of positions was considered in contact if a pseudoatom located at the mean coordinates of one position’s sidechain atoms was less than 6 Å from the corresponding pseudoatom of the other position. For the Pfam dataset, a pair of positions in an MSA of a protein family was considered to be a true contact if the corresponding pair of positions was in contact within any PDB structure mapped to the family. For the CASP12 dataset, a pair of positions in an MSA was considered to be a true contact if the corresponding pair of positions was in contact in the PDB structure of the target sequence. To enable direct comparison between the results in this paper and those in [12], a contact in the Pfam dataset was treated as a contact only if the two positions were separated in sequence by at least five positions. On the other hand, a contact in the CASP12 dataset was treated as a contact only if the two positions were separated in sequence by at least six positions, in accordance with CASP protocol (see http://predictioncenter.org/casp12/doc/rr_help.html).

### 4.7 Contact prediction

To predict contacts, all residue pairs separated by at least the minimum sequence separation (see the previous paragraph for details) were ranked in descending order of calculated co-evolution scores and top-ranking pairs were predicted as contacting. Top pairs were selected either based on a fixed rank cutoff (i.e., the first *N* pairs predicted as contacting for each protein, as in Figs 3A, 3C, and 4) or a length-normalized rank cutoff (i.e., for a protein of length *N*, the first *f* × *N* pairs predicted as contacting, with *f* ∈ [0, 1], as in Fig 3B and 3D). Positive predictive value (PPV) was assessed as the fraction of true contacts out of the predicted contacts. Since the set of true contacts depends on the geometric contact definition, PPV was a function of contact definition.

## Supporting information

S1 Data Protein family alignments. Each file in the ‘alignments’ directory herein corresponds to a protein family’s alignment and contains the accession number and sequence range of each sequence in the alignment.
(TAR.GZ)

S2 Data PDB dataset. A text file containing the PDB ID and chain ID of each structure used in the construction of contact potentials.
(TXT)

S3 Data Contact potentials. Each contact potential is stored as a CSV file, wherein each line specifies the energy for a pair of amino acids. The files are named according to the contact potential they encode, e.g. cp-1.csv is the contact potential for definition 1 in Table 1.
(TAR.GZ)

S1 Fig Average PPV of contact prediction as a function of sequence separation. Average PPV of contact prediction as a function of the number (N) of predictions broken down by the sequence separation of the contacts. Predictions labeled by CD refer to predictions when contacts are defined by contact degree and those labeled by C1, C2, and C3 refer to predictions when contacts are defined by the other three definitions (see Table 1 for details). Contacts are partitioned into three categories based on sequence separation: (A, B) short-range (6 ≤ sequence separation ≤ 11); (C, D) medium-range (12 ≤ sequence separation ≤ 23); (E, F) long-range (23 ≤ sequence separation). Plots A, B, and E depict the predictions of DCA on the Pfam dataset. Plots B, C, and F depict the predictions of MetaPSICOV on the CASP12 dataset.
(PDF)

S2 Fig Performance of DCA vs CD-based contact potential alone. DCA performance on the Pfam dataset compared to the performance of the CD-based contact potential alone. Predictions labeled by DI refer to DCA’s predictions without the incorporation of a contact potential and those labeled by CD refer to the predictions made using the contact potential alone.
(PDF)

S1 Script DCA script. A MATLAB script written by Morcos *et al.* that computes direct information.
(M) Click here for additional data file.
