# Supplementary figures and images for: Contact prediction is hardest for the most informative contacts, but improves with the incorporation of contact potentials
Source: PLoS One. 2018 Jun 28;13(6):e0199585. doi: 10.1371/journal.pone.0199585 (PMC6023208; doi:10.1371/journal.pone.0199585)

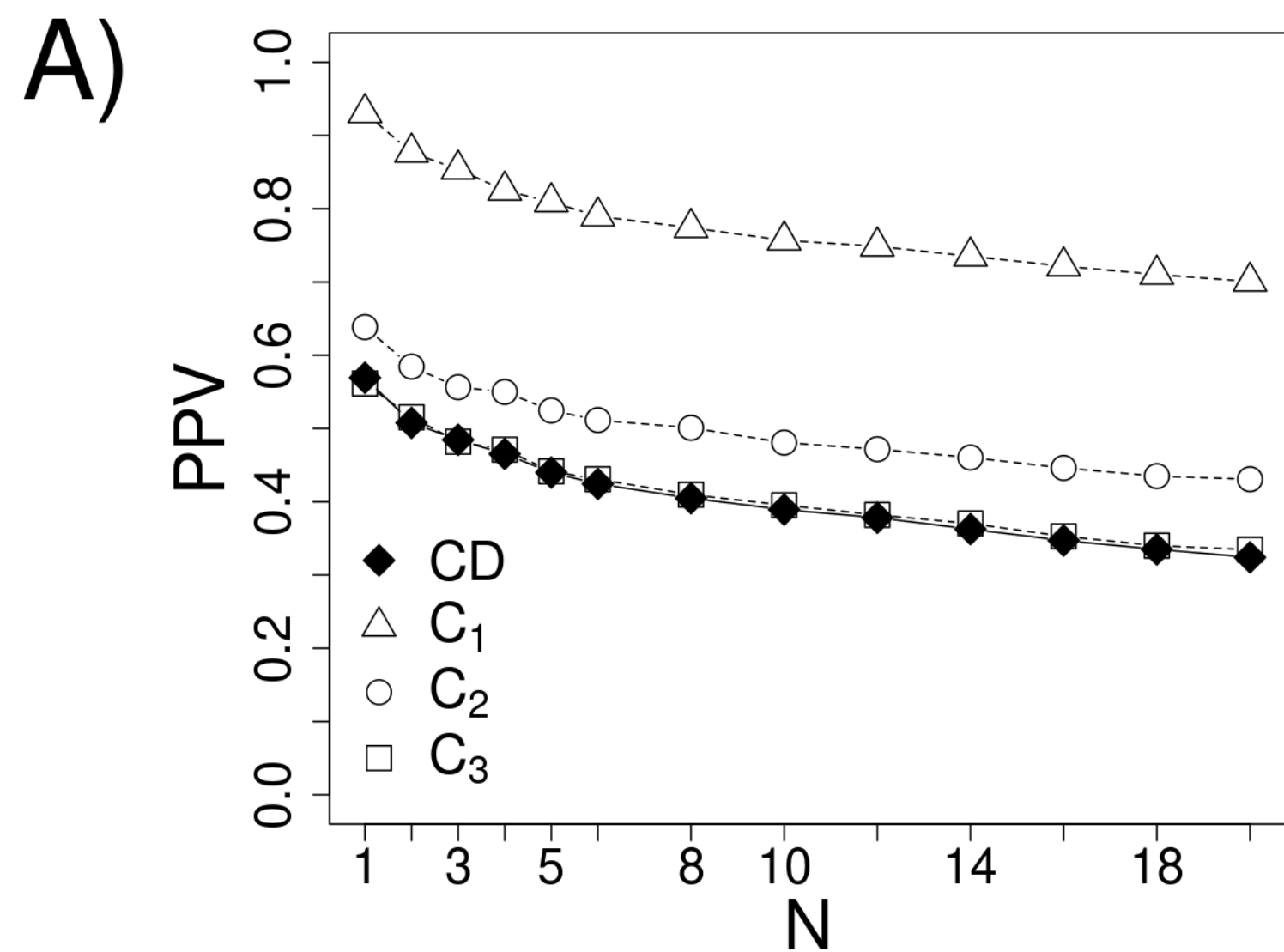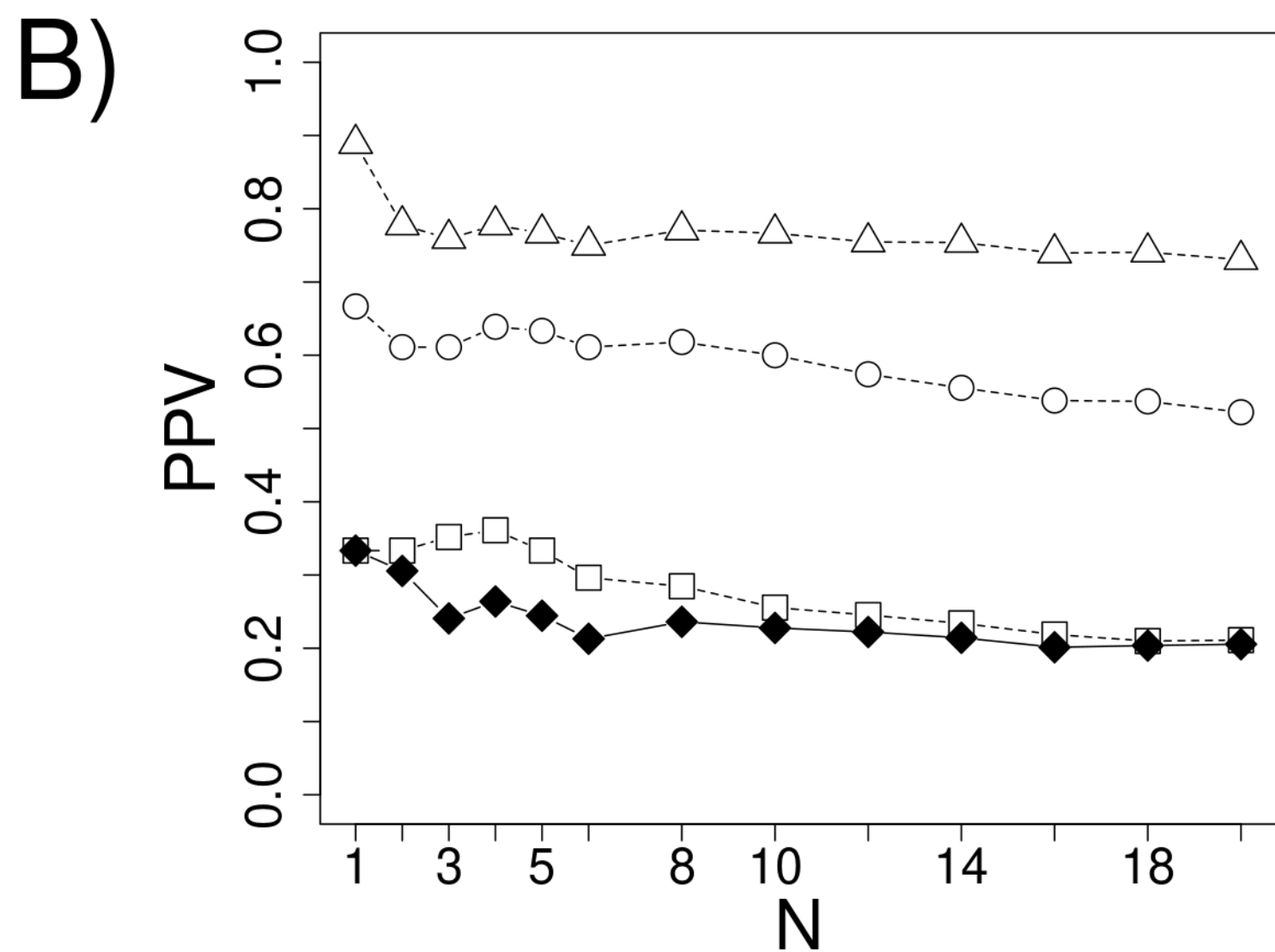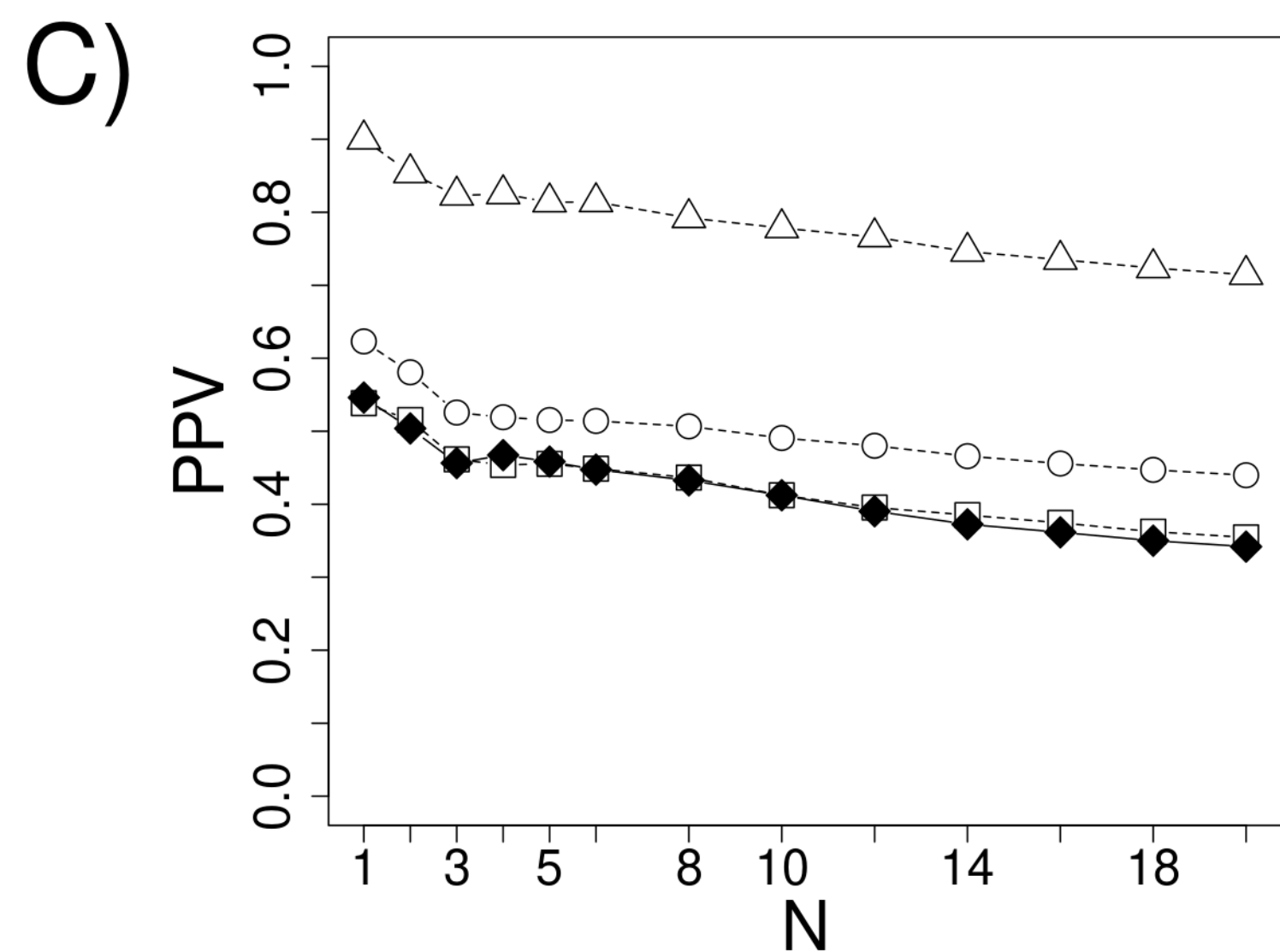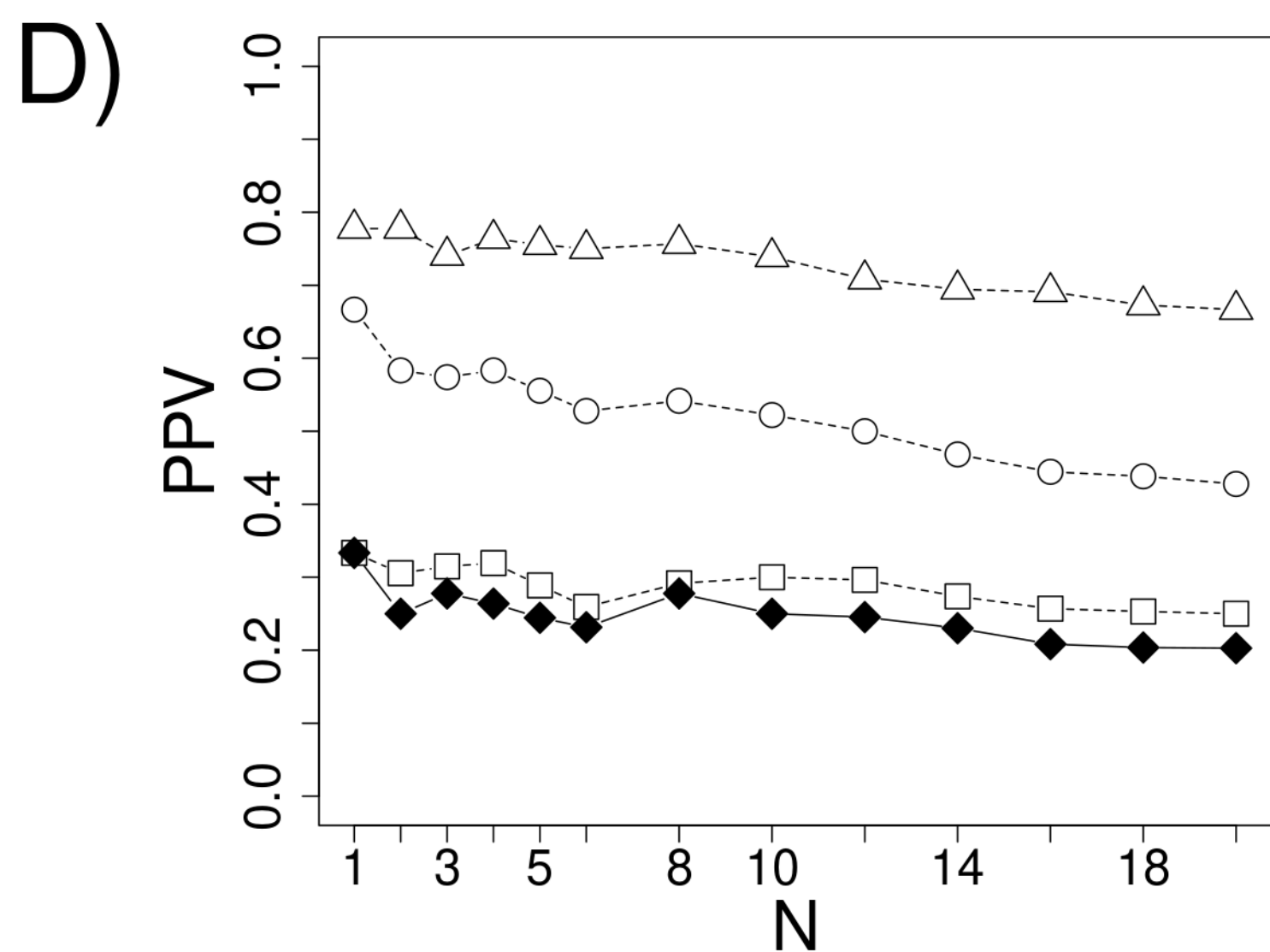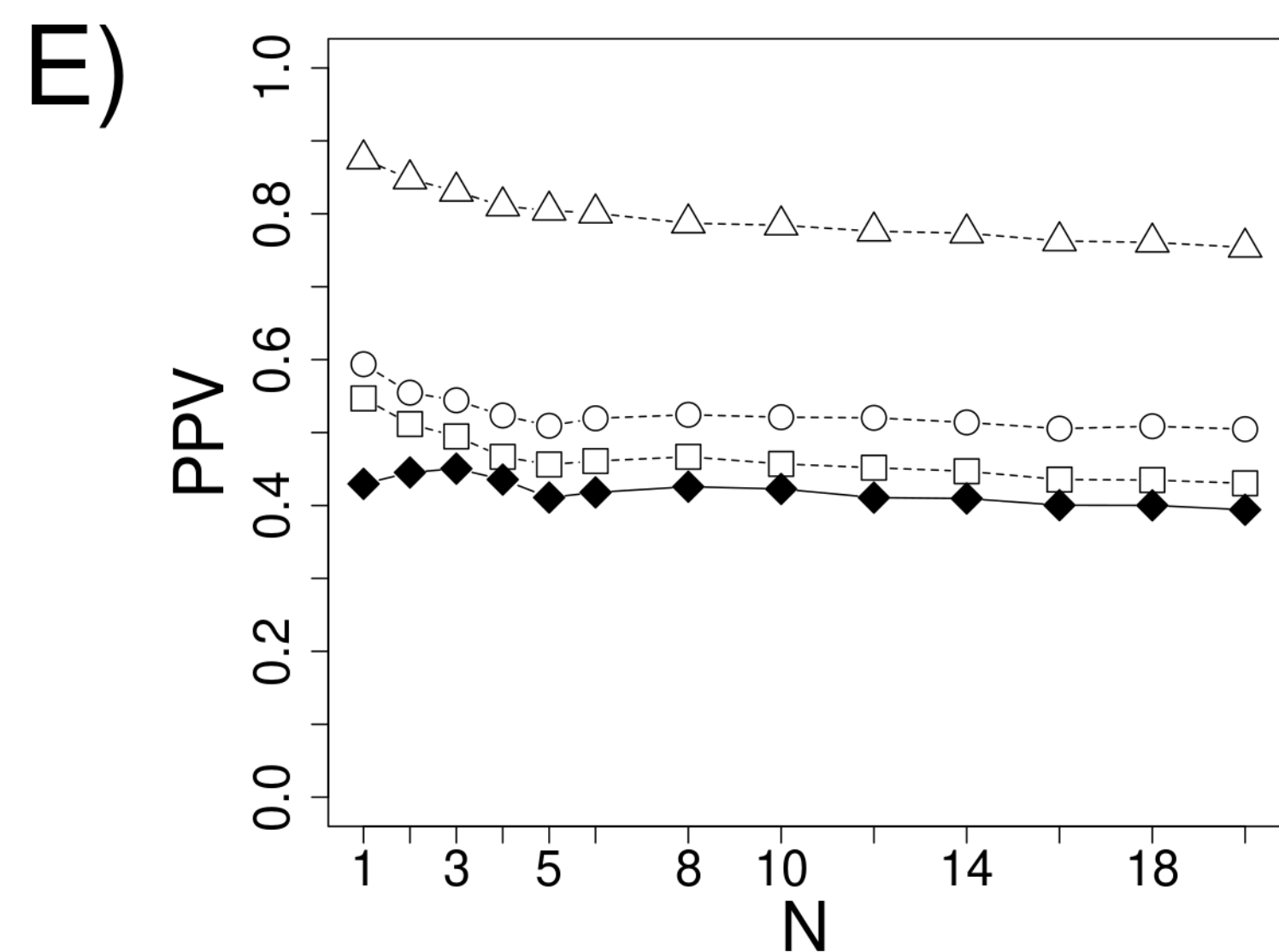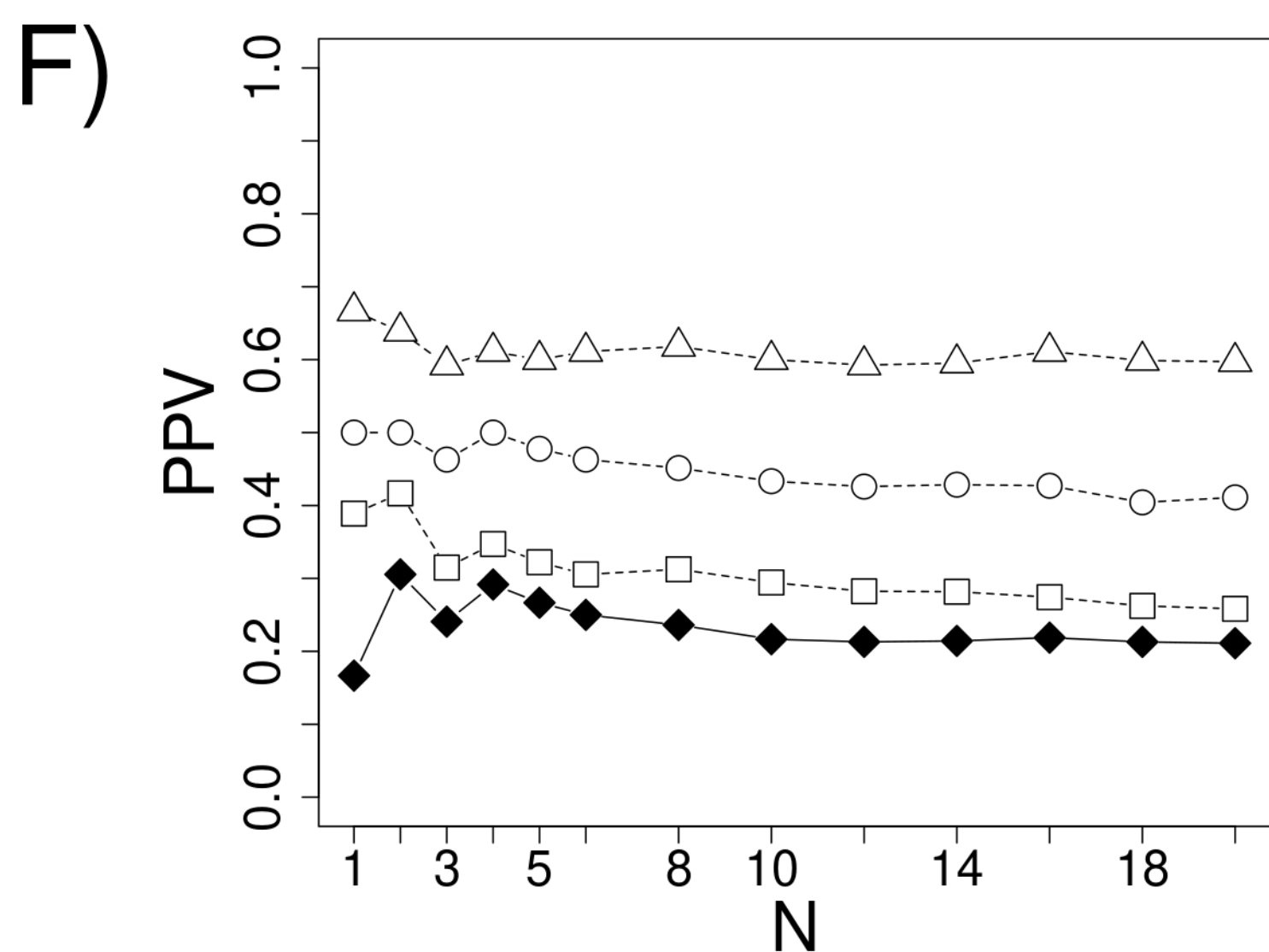

Supplement: S1 Fig — Average PPV of contact prediction as a function of the number (N) of predictions broken down by the sequence separation of the contacts. Predictions labeled by CD refer to predictions when contacts are defined by contact degree and those labeled by C1, C2, and C3 refer to predictions when contacts are defined by the other three definitions (see Table 1 for details). Contacts are partitioned into three categories based on sequence separation: (A, B) short-range (6 ≤ sequence separation ≤ 11); (C, D) medium-range (12 ≤ sequence separation ≤ 23); (E, F) long-range (23 ≤ sequence separation). Plots A, B, and E depict the predictions of DCA on the Pfam dataset. Plots B, C, and F depict the predictions of MetaPSICOV on the CASP12 dataset. (PDF) [file pone.0199585.s004.pdf]

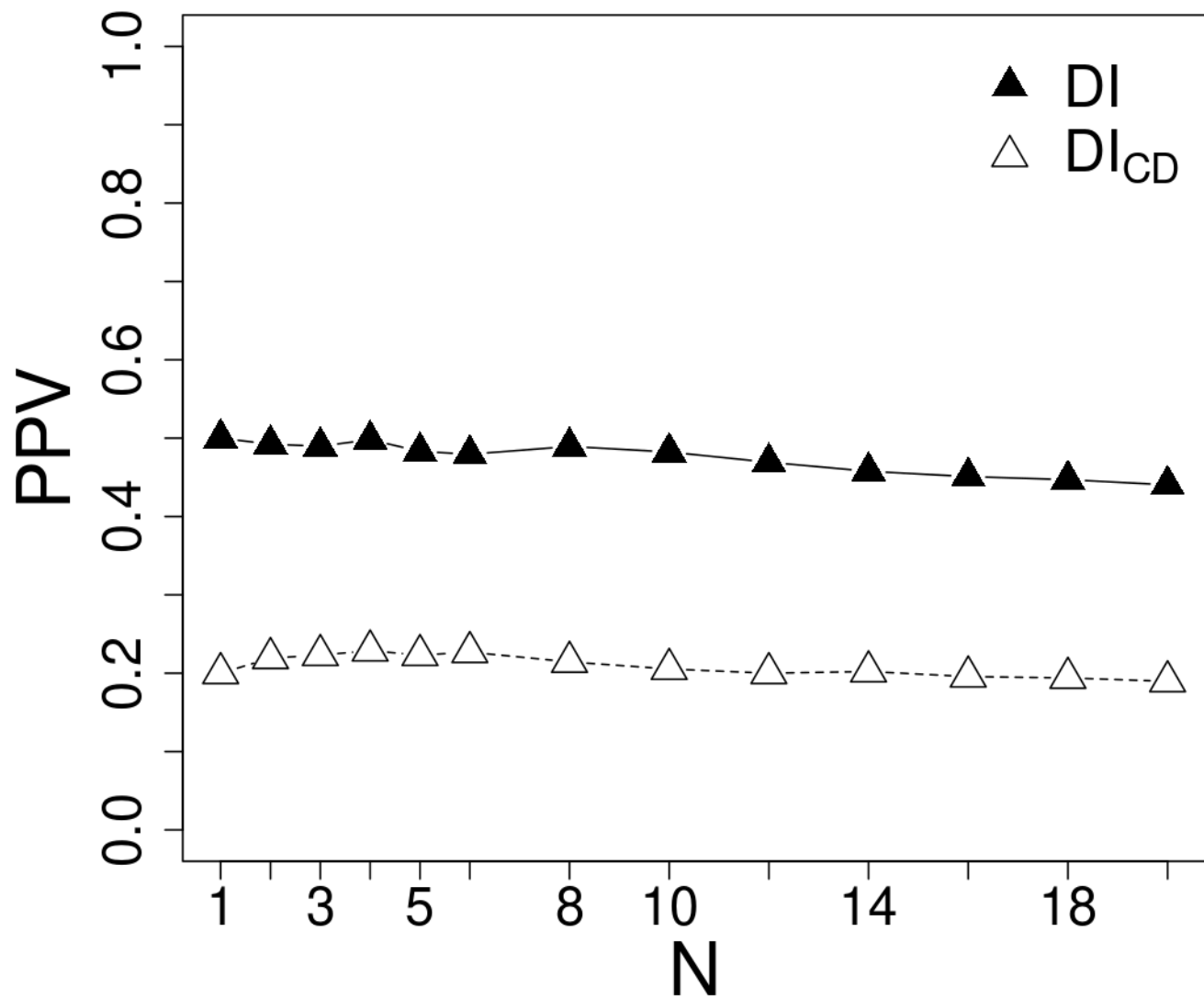

Supplement: S2 Fig — DCA performance on the Pfam dataset compared to the performance of the CD-based contact potential alone. Predictions labeled by DI refer to DCA’s predictions without the incorporation of a contact potential and those labeled by CD refer to the predictions made using the contact potential alone. (PDF) [file pone.0199585.s005.pdf]
